# Publishing a pandemic

**DOI:** 10.1038/s41467-022-33051-z

**Published:** 2022-09-20

## Abstract

Over the last two and a half years, *Nature Communications* has received thousands of submissions related to the COVID-19 pandemic and accepted hundreds for publication. To showcase the breadth and quality of this work, we are now launching a COVID-19 Collection, and here we reflect on our editorial processes during this period.

Keeping up with the pace and volume of scientific research during the pandemic has presented a significant editorial challenge. There has been a genuine need to ensure that important findings across a range of fields are peer-reviewed and made available to a global audience as rapidly as possible. *Nature Communications* is well-placed to facilitate this owing to our multidisciplinary scope, open access model, and system of continuous online publication. However, we have also had to ensure that speed has not come at the cost of publishing standards.

As always, peer reviewers have played a vital role in ensuring the quality of the submissions that we accept for publication. We are very grateful to the many hundreds of scientists who provided insightful peer review reports with faster than usual deadlines during the pandemic. To reduce duplication of reviewer effort, we have promoted our portable peer review policy, which allows papers rejected at other journals to be submitted with their peer review reports. Most often, this involves transfer between journals within the Nature Portfolio, but we also encourage authors to make this request when submitting work previously reviewed at external publishers willing to engage with the portable peer review concept, in order to save time and resources.

“We are very grateful to the many hundreds of scientists who provided insightful peer review reports with faster than usual deadlines during the pandemic.”

The rapid pace of COVID-19 research has inevitably meant that different research groups have been working on the same scientific questions simultaneously. We have supported these researchers by applying our ‘scoop-protection’ policy that allows us to consider multiple papers on similar topics or that reproduce results published elsewhere when received within a short timeframe. In further recognition of the importance of reproducibility and transparency, we have recently tightened our policies on the availability of data, code, materials and associated protocols. We also continue to encourage our authors to accept the option to publish peer review reports alongside the final version of their article, adding clarity to our editorial decisions.

Our new Collection highlights some of the most impactful and innovative work that we have published across the spectrum of COVID-19 research, including papers in immunology, vaccines, pathology, therapy, epidemiology, public health, and molecular and cell biology. We also have a Clinical Collection, that presents articles, including results from clinical trials relating to COVID-19, in this growing area for the journal. There is, however, still much to be understood about the SARS-CoV-2 virus and COVID-19 disease. Emerging topics that we hope our journal can contribute to in the coming months include:

Improvements to vaccines and therapies (such as newly designed antigens, different formulations or adjuvants, and alternative administration routes that could extend the breadth and durability of the immune response);

Better understanding of host-pathogen interactions (for example, elucidating the underlying mechanisms driving post COVID-19 condition through development of novel experimental models, and determining functions of viral proteins);

Estimating impacts of COVID-19 interventions on other infectious diseases (including their epidemiology, symptoms, and severity, which may involve mathematical modelling, improved surveillance, pathogen genomics, and multiplexed serology).

*Nature Communications* continues to welcome all submissions relating to the COVID-19 pandemic as we find our way out of it.

